# Multiple Oral and Eyelid Nodules in a Pediatric Patient

**DOI:** 10.1111/odi.70187

**Published:** 2026-04-21

**Authors:** Caique Mariano Pedroso, Paulo Victor Mendes Penafort, Patrícia Vitor de Souza, Fernanda M. Pascon, Regina Maria Punppin‐Rontani, Beatriz Frolini Paulo Cherve, Rachel Marinho Robim, Regina Maria Holanda de Mendonça, Marcio Ajudarte Lopes, Pablo Agustin Vargas, Alan Roger Santos‐Silva

**Affiliations:** ^1^ Piracicaba Dental School, State University of Campinas Piracicaba Brazil; ^2^ IAP, Laboratory of Pathology Anatomy Piracicaba São Paulo Brazil; ^3^ Department of Dentistry Boldrini Hospital Campinas São Paulo Brazil

**Keywords:** diagnosis, MEN2B, oral mucosal nodules

## 1 Case Report

**FIGURE 1:**
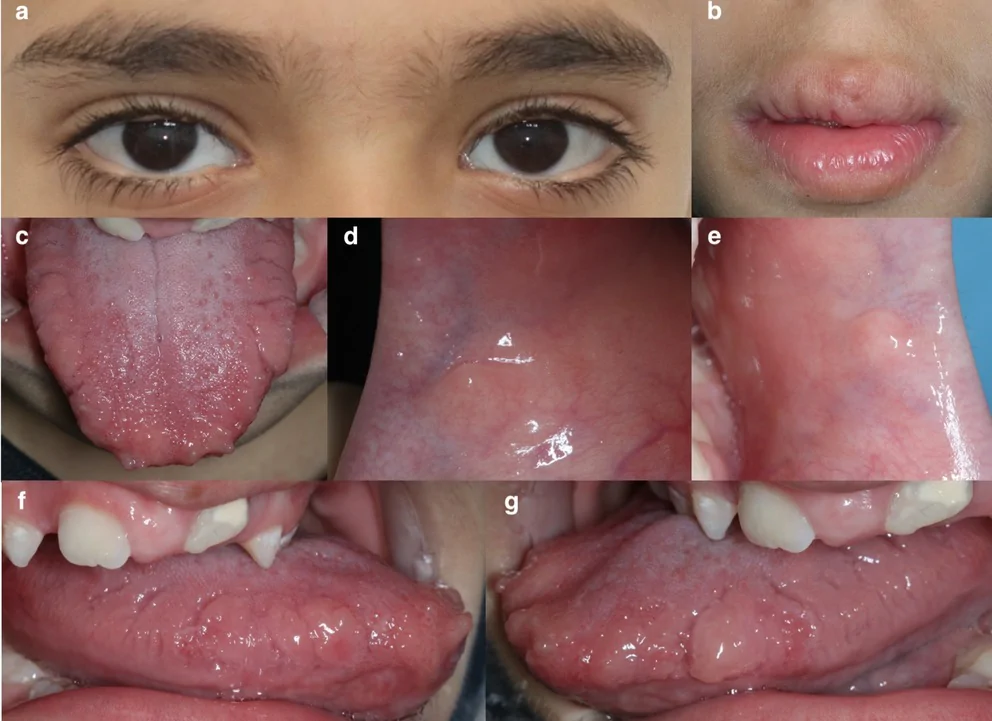
Clinical presentation of nodular lesions. (a) Nodular lesions on the right and left eyelids. (b) Multiple well‐defined, yellowish fibrous nodules on the upper lip causing mild protrusion. (c) Enlarged tongue with multiple nodules on the tip and anterior one‐third. (d–e) Nodular masses on the right and left buccal mucosa. (f–g) Intraoral nodules that are asymptomatic, oval to round, sessile, and smooth‐surfaced.

An 8‐year‐old male was referred to the oral medicine service due to the presence of multiple nodules on his upper lip, tongue, and buccal mucosa. The parents stated that the patient had exhibited tongue alterations since birth and, over the past 2 months, they had noticed abnormal growth in his lip. There was no family history of cancer or thyroid abnormalities. On extraoral examination, nodular lesions were observed on both the right and left eyelids (Figure 1a). Additionally, multiple well‐defined, yellowish fibrous nodules were noted on the upper lip, resulting in a mild protrusion relative to the lower lip (Figure 1b). The tongue appeared enlarged on intraoral examination, with multiple nodules on its tip and anterior one‐third (Figure 1c). Similar nodular masses were also present on the right and left buccal mucosa (Figure 1d,e). The intraoral nodules were asymptomatic, oval to round in shape, had a sessile base, and exhibited a smooth surface (Figure 1f,g). An incisional biopsy of a tongue nodule was performed, and laboratory and imaging exams were requested.

## 2 What is Your Diagnosis?

Based on the patient's history, physical examination, and laboratory findings, which one of the following is the most suspicious diagnosis?

1. Orofacial granulomatosis
2. Oral Amyloidosis
3. Multiple Endocrine Neoplasia type 2B
4. Neurofibromatosis

## 3 Diagnosis

**FIGURE 2:**
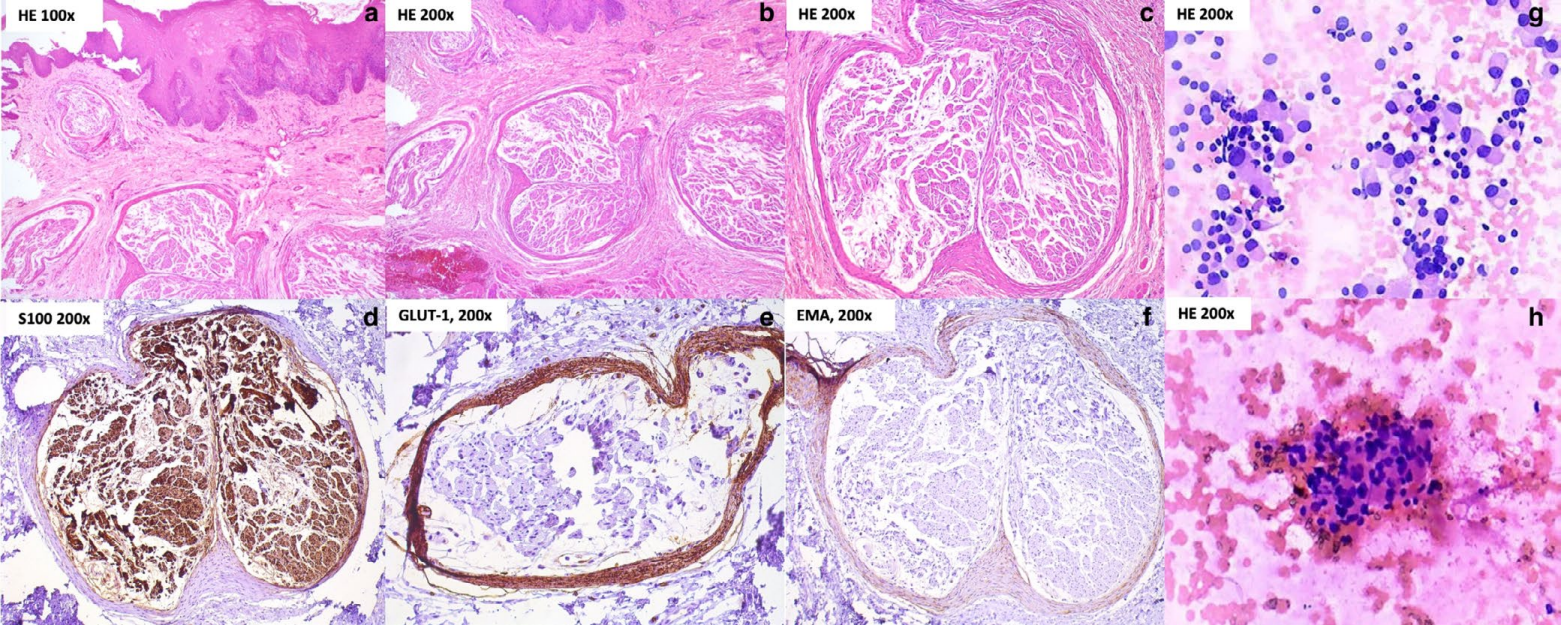
Histopathological and cytological findings. (a–c) Hematoxylin and eosin–stained sections showing hyperplastic nerve bundles with loose connective tissue and marked perineurium thickening. (d) Immunohistochemistry for S‐100 protein confirming the neural origin of the lesion. (e–f) Glut‐1 and EMA positivity highlighting perineurium thickening. (g–h) Fine‐needle aspiration cytology of the thyroid showing isolated cells and syncytial‐like clusters of plasmacytoid, polygonal, and round cells with marked pleomorphism, consistent with medullary thyroid carcinoma.

The patient diagnosis was multiple endocrine neoplasia type 2B (MEN2B). Histopathological sectional stained with H&E showed hyperplastic nerve bundles with loose connective tissue and prominent thickening of the perineurium (Figure 2a–c). Immunohistochemistry for S‐100 protein confirmed the neural origin of the lesion (Figure 2d). Glut‐1 (Figure 2e) and EMA (Figure 2f) positivity highlighted the perineurium thickening. Laboratory investigations revealed hypocalcemia (8.9 mg/dL), elevated carcinoembryonic antigen (13.7 ng/mL), and markedly increased calcitonin levels (818 pg/mL). Parathyroid hormone (29.0 pg/mL) levels were within the reference range. Ultrasonography revealed two irregular nodules in the thyroid gland: one measuring 0.4 × 0.4 cm located in the middle third of the right lobe, and another measuring 0.7 × 0.6 cm in the middle third of the left lobe, both classified as TI‐RADS 5, suggesting a high suspicion of malignancy. Subsequently, fine‐needle aspiration cytology of the thyroid was performed, revealing numerous isolated cells and syncytial‐like clusters composed of plasmacytoid, polygonal, and round cells exhibiting marked cellular pleomorphism, consistent with a diagnosis of medullary thyroid carcinoma (Figure 2g,h). Based on clinical, histopathological, laboratory, imaging, and cytology findings, a diagnosis of MEN2B was established.

MEN is a rare autosomal dominant hereditary cancer syndrome caused by missense gain‐of‐function mutations in the RET proto‐oncogene on chromosome 10 (Pasquali et al. 2012). First described in 1963, MEN was classified into type 1 (MEN1) and type 2 (MEN2) in 1968 (Wermer 1963; Steiner et al. 1968). MEN2B is the most aggressive subtype, primarily characterized by the early onset of medullary thyroid carcinoma (MTC), pheochromocytomas, and mucosal neuromas (MacIntosh et al. 2014). The syndrome has an estimated incidence of 1 in 1,000,000 live births, with nearly 100% penetrance for MTC, and recognition of oral mucosal is essential to prompt diagnosis as well as better prognosis MTC‐related (Norton et al. 2015). This case highlights the importance of recognizing oral mucosal and eyelid neuromas as an early sign of MEN2B.

Mucosal neuromas are one of the earliest and most distinctive features of MEN2B, often appearing in childhood before systemic manifestations. These nodules most commonly occur on the tongue, buccal mucosa, gingiva, and lips, as well as around the eyelids and conjunctiva (MacIntosh et al. 2014). These lesions are often misdiagnosed as benign conditions, delaying the identification of the syndrome. This hypothesis is confirmed when the patients' parents reported that the oral lesions had been present since birth, and that other professionals say that it is a normal condition. Despite their benign nature, their presence should prompt immediate investigation for MEN2B, as early diagnosis allows for improved survival outcomes by preventing the progression of MTC (Norton et al. 2015). The investigation of MTC can be supported by measuring serum levels of calcitonin and carcinoembryonic antigen, as well as plasma concentrations of catecholamines, including epinephrine and norepinephrine. Laboratory and imaging findings are critical in establishing the diagnosis and are closely associated with the early detection of MTC.

The oral clinical presentation of MEN2B contrasts with other conditions exhibiting similar oral nodules findings. In orofacial granulomatosis, oral manifestations include persistent lip swelling and a cobblestone appearance of the buccal mucosa, often accompanied by regional lymphadenopathy (Troiano et al. 2015). Oral amyloidosis may present with macroglossia, firm nodules, and petechiae, reflecting extracellular deposition of amyloid protein (Pontes et al. 2023). Neurofibromatosis type 1 may present with intraoral neurofibromas, which are generally larger, more irregular, and associated with cutaneous neurofibromas and Lisch nodules (Javed et al. 2014). Therefore, a detailed clinical assessment, supported by histopathology and laboratory testing when appropriate, is crucial to distinguish MEN2B mucosal neuromas from these overlapping conditions and ensure timely diagnosis and management.

## 4 Outcome

Following the diagnosis of MEN2B, the patient was referred to a pediatric oncology center. Locoregional metastases were confirmed, and the patient subsequently underwent surgery for thyroidectomy and lymph node dissection. The patient is under regular follow‐up.

## Author Contributions

**Marcio Ajudarte Lopes:** validation, visualization, writing – review and editing. **Regina Maria Holanda de Mendonça:** validation, visualization, writing – review and editing. **Alan Roger Santos‐Silva:** conceptualization, investigation, methodology, validation, visualization, writing – review and editing, supervision. **Pablo Agustin Vargas:** validation, investigation, visualization, writing – review and editing. **Patrícia Vitor de Souza:** writing – review and editing, visualization, validation, investigation. **Paulo Victor Mendes Penafort:** validation, visualization, methodology, data curation, investigation. **Rachel Marinho Robim:** methodology, validation, visualization, writing – review and editing, investigation. **Beatriz Frolini Paulo Cherve:** validation, methodology, visualization, writing – review and editing, investigation. **Regina Maria Punppin‐Rontani:** validation, visualization, writing – review and editing. **Fernanda M. Pascon:** validation, visualization, writing – review and editing. **Caique Mariano Pedroso:** conceptualization, investigation, writing – original draft, methodology, validation, visualization, writing – review and editing, data curation.

## Consent

The patient reported in this manuscript provided written informed consent for the publication of the case details.

## Conflicts of Interest

The authors declare no conflicts of interest.

## Data Availability

The data that support the findings of this study are available from the corresponding author upon reasonable request.
